# Ecological feedbacks stabilize a turf-dominated ecosystem at the southern extent of kelp forests in the Northwest Atlantic

**DOI:** 10.1038/s41598-019-43536-5

**Published:** 2019-05-08

**Authors:** Colette J. Feehan, Sean P. Grace, Carla A. Narvaez

**Affiliations:** 1 Department of Biology, Montclair State University, Montclair, NJ 07043 USA; 2 Department of Biology and Werth Center for Coastal and Marine Studies, Southern Connecticut State University, New Haven, CT 06515 USA; 3 Department of Biology, Villanova University, Villanova, PA 19085 USA

**Keywords:** Climate-change ecology, Biooceanography

## Abstract

Temperate marine ecosystems globally are undergoing regime shifts from dominance by habitat-forming kelps to dominance by opportunistic algal turfs. While the environmental drivers of shifts to turf are generally well-documented, the feedback mechanisms that stabilize novel turf-dominated ecosystems remain poorly resolved. Here, we document a decline of kelp *Saccharina latissima* between 1980 and 2018 at sites at the southernmost extent of kelp forests in the Northwest Atlantic and their replacement by algal turf. We examined the drivers of a shift to turf and feedback mechanisms that stabilize turf reefs. Kelp replacement by turf was linked to a significant multi-decadal increase in sea temperature above an upper thermal threshold for kelp survival. In the turf-dominated ecosystem, 45% of *S. latissima* were attached to algal turf rather than rocky substrate due to preemption of space. Turf-attached kelp required significantly (2 to 4 times) less force to detach from the substrate, with an attendant pattern of lower survival following 2 major wave events as compared to rock-attached kelp. Turf-attached kelp allocated a significantly greater percentage of their biomass to the anchoring structure (holdfast), with a consequent energetic trade-off of slower growth. The results indicate a shift in community dominance from kelp to turf driven by thermal stress and stabilized by ecological feedbacks of lower survival and slower growth of kelp recruited to turf.

## Introduction

Globally, forests of kelp and other habitat-forming brown seaweeds have been replaced by mats of low-lying turf-forming algae, representing a loss of ecosystem structure and biodiversity^1–4^. Declines of habitat-forming kelps in disparate regions over the past 3 decades have been linked to a range of stressors, with ocean warming and eutrophication being the most commonly identified environmental drivers^5^. However, despite localized kelp declines, there is no unifying global pattern of kelp change, with some populations remaining unchanged and others expanding over the past half century^6^. Based on studies at early life-history stages, it is predicted that warm-adapted kelp populations existing at the equatorial range limits of species distributions may be the most resilient to climate change and most likely to persist into the future^7^. Longitudinal field observations at the range limits of kelp species are required to test this hypothesis.

Where regime shifts from kelp to turf dominance have occurred, various feedback mechanisms may stabilize the turf-dominated ecosystem state contributing to its temporal persistence^5^. Such feedbacks include high sediment loads, kelp spore limitation, and space limitation on turf reefs, which prevent recruitment and reestablishment of kelps^8–10^. However, in general, information is scant on the ecological functioning of novel turf-dominated ecosystems^11^. In particular, it is unknown whether the turf-dominated state represents an alternative stable state (sensu Scheffer *et al*. ref.^12^) of the kelp ecosystem, with both kelp- and turf-dominated states occurring under the same set of environmental conditions (i.e. with hysteresis)^5^. A better theoretical ecological understanding of regime shifts to turf will improve our ability to predict the reversibility of these shifts once an initial environmental driver (e.g., eutrophication) is relaxed.

At the southern extent of kelp forests in the Northwest Atlantic, the dominant kelp species *Saccharina latissima* exists near its upper thermal threshold for survival^13^, providing a useful model for examining the resilience of warm-adapted kelp forests. Peak recruitment in this region occurs in the winter and spring following sporogenesis in early autumn and spring^14,15^. The life history of *S. latissima* alternates between annual and biennial growth, with annual cohorts occurring in the warmest years due to blade degradation in the late summer and low survival into the following year^16^. This is in contrast to more northerly locations in the North Atlantic where populations of *S. latissima* are perennial with individual lifespans of 2–3 years^17,18^. The macroscopic diploid sporophyte stage of *S. latissima* has a lower thermal tolerance than the microscopic haploid gametophyte stage^15^. Laboratory and field studies have indicated that at the southern extent, the gametophyte stage can persist through supraoptimal summer temperatures, acting as a warm-tolerant oversummering “resting stage” as an adaptation to dealing with warm temperatures^15^.

Surprisingly, the most recent quantitative data of kelp abundance (biomass and density) for this region are from the 1980s^16,19^. At that time, kelp forests in Rhode Island Sound and Long Island Sound, USA had primary productivity similar to that of more northerly populations in Nova Scotia, Canada, and the United Kingdom, with standing biomass of up to 24 kg m^−2^ ^16,19^, indicating healthy kelp forests at the southern extent. However, mean sea surface temperatures over ~6 decades from 1948 to 2012 warmed at the southern extent at a rate of 0.3 °C decade^−1^ ^20^, with unknown impacts on kelp populations.

In fall 2017, divers observed low abundance of kelp at a site (Fort Wetherill) at the mouth of Narragansett Bay, Rhode Island that was previously dominated by a dense *S. latissima* kelp forest^19^. Young *S. latissima* sporophytes that were present were largely attached to turf-forming macroalgae rather than rocky substrate, a phenomenon that was previously rare^19^. The observations prompted us to conduct a field study to evaluate the state of kelp forests at the southern extent with 4 main objectives: (1) to quantify kelp biomass and density for comparison with available baseline data from the 1980s; (2) to determine whether algal turf has replaced kelp as the dominant benthic primary producers; (3) to examine correlative evidence for the environmental drivers of an apparent decline of kelp and the rise of turf; and (4) to elucidate the feedback mechanisms that stabilize a turf-dominated ecosystem; specifically, by examining the ecological consequences of kelp recruiting onto turf (e.g., morphology, growth, and survival of kelp on turf versus rocky substrate). To determine whether long-term ocean warming is a likely driver of kelp decline, we analyzed weekly sea surface temperature data from the Narragansett Bay Plankton Time Series collected over a 58-year period from 1959 to 2017 (see Methods). Based on previous observations that the rate of erosion of the distal end of *S. latissima* sporophytes exceeds the rate of meristematic growth at temperatures above 20 °C^14^, and that 22 °C is an upper critical limit for *S. latissima* gametophyte survival^21^, we developed a thermal integral of weeks with sea temperatures ≥22 °C (degree-week) for each year as an indication of the magnitude of kelp thermal stress through time. Given that eutrophication also is cited as a common cause of a regime shift from kelp to turf (e.g., in Russia, Denmark, Norway, Brazil, and Australia)^5^, we also examined time series of ammonium, nitrate, and phosphate concentrations from the Narragansett Bay Plankton Time Series collected over a 46-year period from 1972 to 2018.

## Results

### Decline of kelp at the southern extent

**Figure 1:**
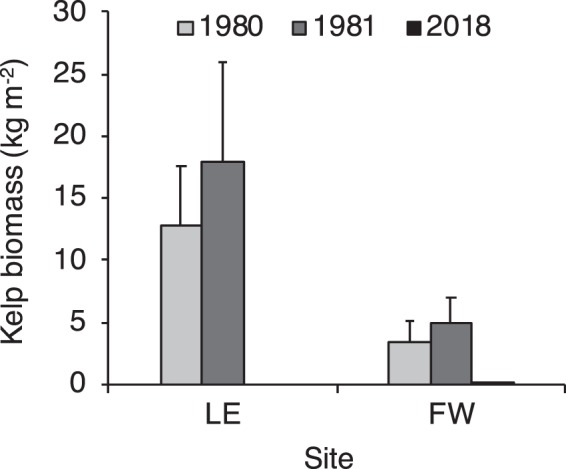
Mean kelp biomass (kg m^−2^) at sites in Narragansett Bay (Land’s End, LE; and Fort Wetherill, FW) in 1980 and 1981 from Brady-Campbell *et al*. (ref.^19^) and 2018 from this study. Biomass in 2018 is zero at LE and 0.114 ± 0.063 kg m^−2^ at FW (only partially visible on graph). Error bars are SE for n = 6 quadrats per year.

**Figure 2:**
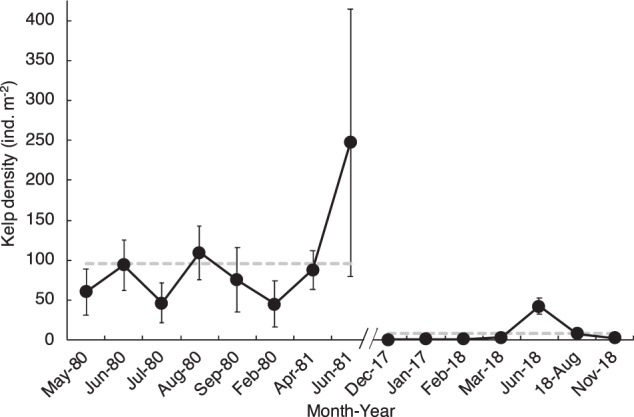
Mean kelp density (ind. m^−2^) at Fort Wetherill from 1980–81 in Brady-Campbell *et al*. (ref.^19^) and 2017–18 in this study. Error bars are SE for n = 2–3 belt transects or 10–20 quadrats (some errors within the diameter of the symbol). The gray horizontal dashed lines indicate mean densities from 1980 to 1981 and 2017 to 2018.

Kelp biomass measured at 2 sites in Narragansett Bay (Land’s End and Fort Wetherill) was at least an order of magnitude lower than the biomass observed in the early 1980s (Fig. 1). At Land’s End, mean biomass in summer 2017 was 0 ± 0 kg m^−2^, as compared to a biomass in summer 1980 and 1981 of 12.7 ± 4.88 kg m^−2^ and 17.9 ± 7.97 kg m^−2^, respectively (±SE; n = 6 quadrats). At Fort Wetherill, mean biomass in summer 2017 was 0.114 ± 0.063 kg m^−2^, as compared to a biomass in summer 1980 and 1981 of 3.47 ± 1.69 kg m^−2^ and 4.86 ± 2.09 kg m^−2^, respectively (±SE; n = 6 quadrats). Accordingly, mean kelp density measured at Fort Wetherill over a 1-year period from December 2017 to November 2018 (8.84 ± 5.69 ind. m^−2^) was an order of magnitude lower than a baseline kelp density measured at this site over 1 year from May 1980 and June 1981 (95.8 ± 23.1 ind. m^−2^) (±SE; n = 7 and 8 sampling months in 2017/18 and 1980/81, respectively) (Fig. 2).

### Dominance of a kelp ecosystem by algal turf

**Figure 3:**
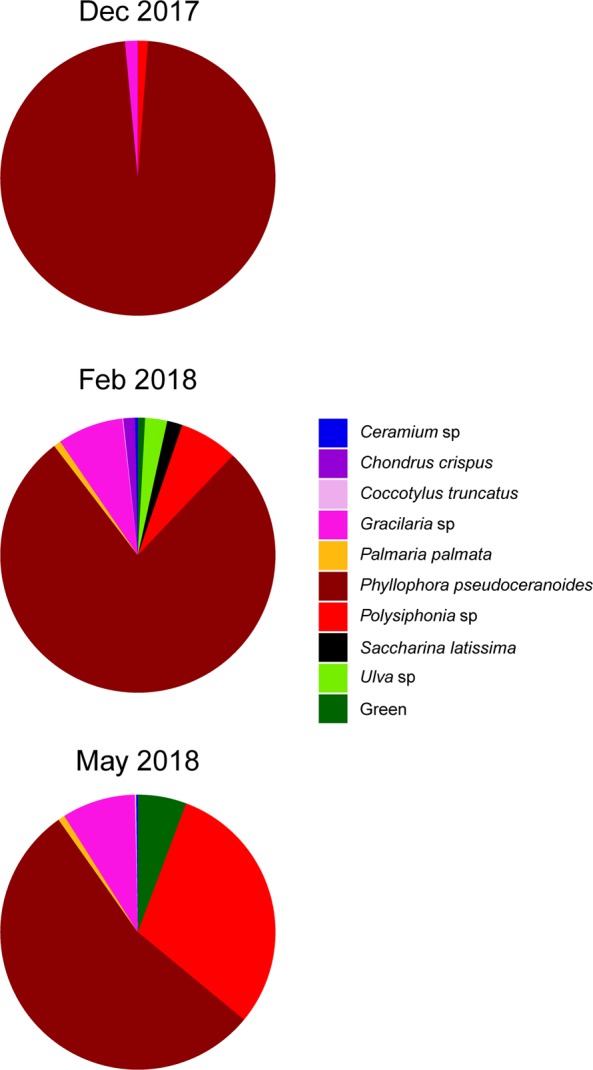
Macroalgal species composition (% wet weight) at Fort Wetherill in winter 2017 and spring 2018.

Rocky reefs at both sites were dominated by turf-forming algae in summer 2018; mean percent areal cover of turf was 91.9 ± 3.3 and 64.2 ± 7.9% (±SE) at Land’s End and Fort Wetherill, respectively. By contrast, in summer 1990, the rocky substratum in the vicinity of Fort Wetherill and Land’s End was dominated (>50%) by *S. latissima*^22^. A third site sampled (Bass Rock Road) had a baseline kelp percent cover of ~80–100% in the mid 1990s (S. Grace pers. obs.) and had percent cover of turf of 94.1 ± 2.6% in summer 2018. The turf assemblage sampled at Fort Wetherill on 3 dates from winter 2017 to spring 2018 consisted of 8 species of red and green macroalgae (Fig. 3). Based on wet weight measurements, the turf assemblage was dominated by a single species, *Phyllophora pseudoceranoides* (mean ± SE: 78 ± 6%, n = 3 dates), with *Polysiphonia* spp. and *Gracilaria* spp. also appearing quite common and accounting for 11 ± 4% and 6 ± 3% of wet weight, respectively (Fig. 3).

### Environmental drivers of kelp decline

**Figure 4:**
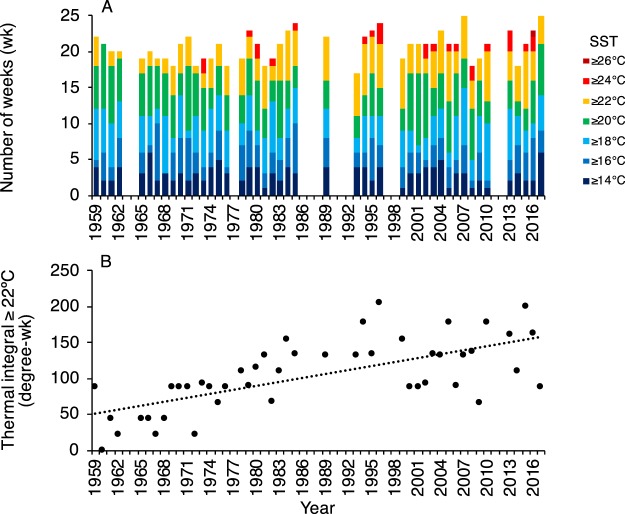
(**A**) Total number of weeks (wk) with sea surface temperatures (SST) greater than or equal to 7 threshold values between 14 °C and 26 °C (2 °C increments), and (**B**) the thermal integral of SST ≥22 °C (degree-wk) for each year from 1959 to 2017. The dotted line indicates a significant linear increase in the thermal integral over time (R^2^ = 0.45; p < 0.001). Data are from ref.^33^ and https://web.uri.edu/plankton/. Gaps in A indicate missing data.

**Figure 5:**
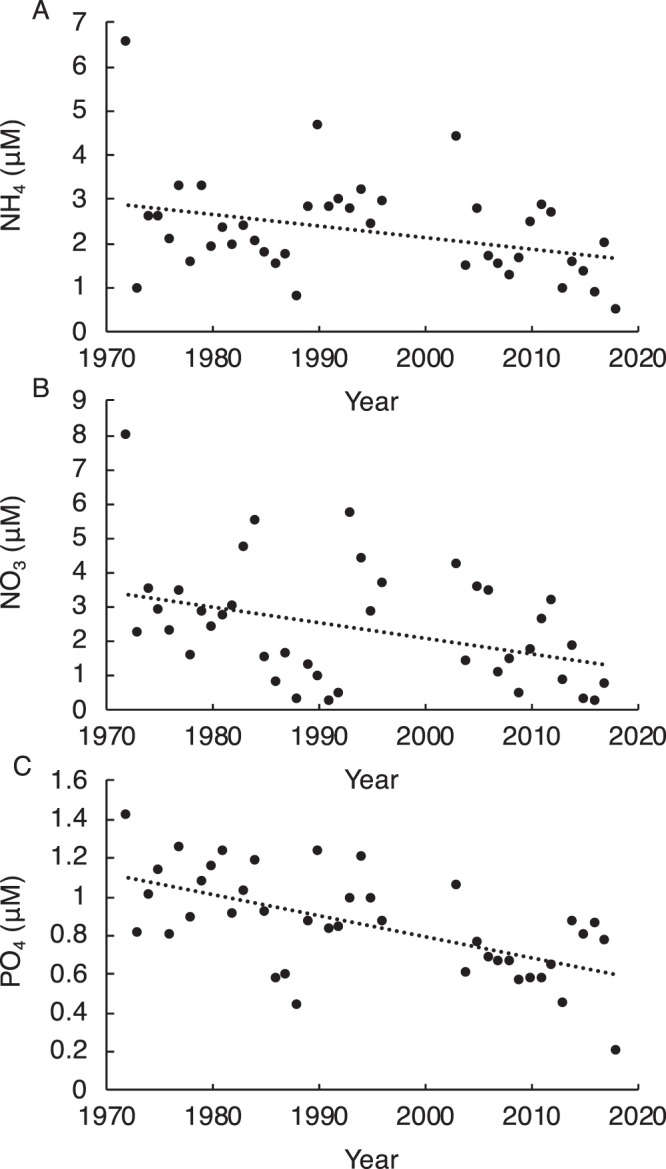
Concentrations (μM) of (**A**) ammonium (NH_4_), (**B**) nitrate (NO_3_), and C. phosphate (PO_4_) measured in Narragansett Bay between 1972 and 2018. The dotted lines indicate significant linear decreases over time (**A**). R^2^ = 0.11; p = 0.033; (**B**) R^2^ = 0.14; p = 0.017; and (**C**) R^2^ = 0.37; p < 0.001). Data are from ref.^33^ and https://web.uri.edu/plankton/.

Consistent with a decline in kelp abundance, weeks with sea surface temperatures ≥22°C were more common through time from 1959 to 2017 (Fig. 4A), with a significant increase in the thermal integral ≥22 °C over this period (Fig. 4B). In contrast, there was a significant decrease in the concentrations of ammonium (NH_4_), nitrate (NO_3_), and phosphate (PO_4_) in Narragansett Bay from 1972 to 2018 (Fig. 5).

### Ecological consequences of kelp recruitment onto turf

**Figure 6:**
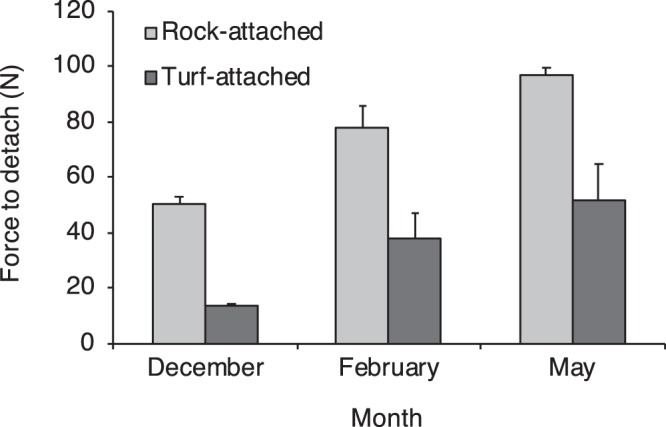
Mean (+SE) force (N) to detach kelp *Saccharina latissima* from rocky substrate (rock-attached) or turf-forming macroalgae (turf-attached) in winter 2017 and spring 2018 (Dec 2017: n = 15 rock, and 12 turf; Feb 2018: n = 5 rock, and 6 turf; May 2018: n = 8 rock, and 9 turf).

**Figure 7:**
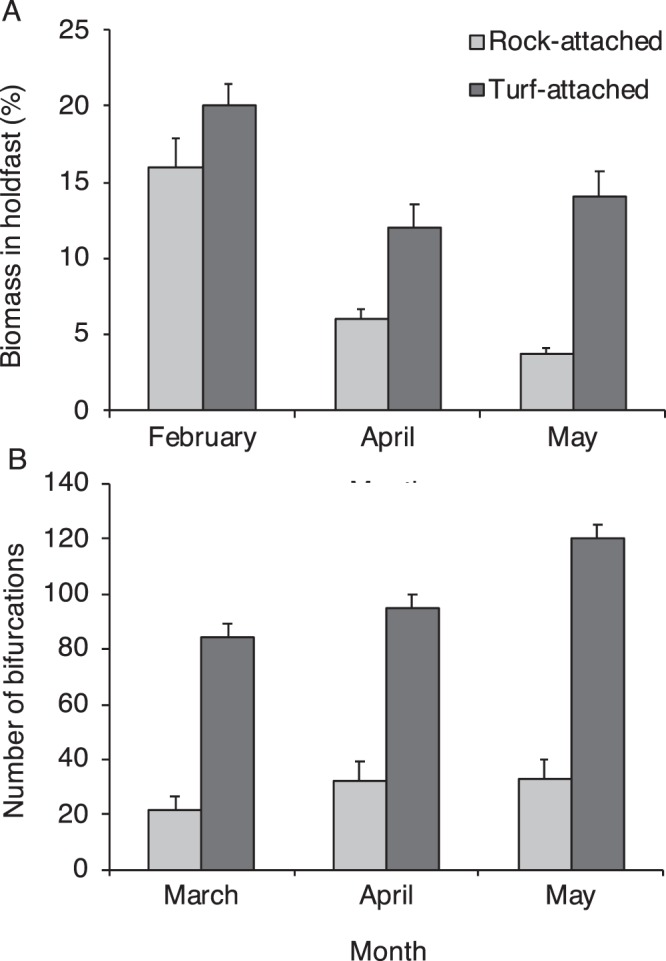
(**A**) Mean (+SE) percentage of biomass (%) allocated to the holdfast of rock-attached and turf-attached kelp *Saccharina latissima* in winter and spring 2018 (Feb: n = 5 rock, and 7 turf; Apr: n = 10 rock, and 10 turf; May: n = 5 rock, and 9 turf). (**B**) Mean (+SE) total number of hapteral bifurcations within the holdfasts of rock-attached and turf-attached kelp (Mar: n = 10 rock, and 9 turf; Apr: n = 10 rock, and 10 turf; May: n = 10 rock, and 9 turf).

**Figure 8:**
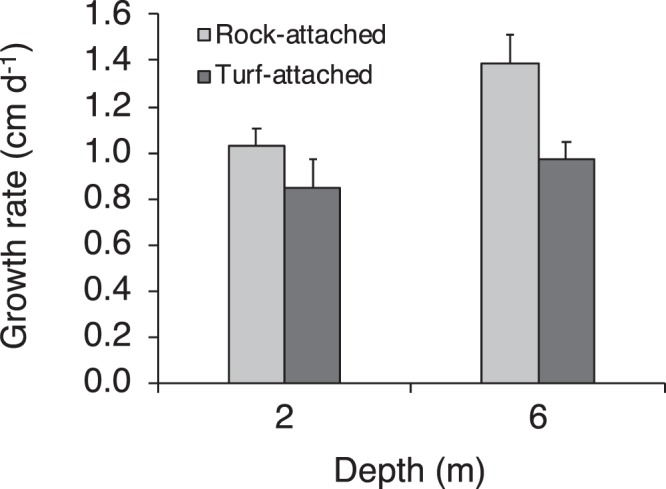
Mean (+SE) growth rate (cm d^−1^) of kelp *Saccharina latissima* on rocky substrate (rock-attached) or turf-forming macroalgae (turf-attached) at 2 m (n = 5 rock, and 5 turf) and 6 m (n = 9 rock, and 3 turf) depth in spring 2018.

At Fort Wetherill from winter 2017 to spring 2018 we observed that on average 45 ± 11% of kelp were attached to turf-forming macroalgae (±SD, n = 4 sampling dates). *S. latissima* attached to turf required significantly (2 to 4 times) less force to remove from the substratum as compared to *S. latissima* attached to rock (Fig. 6, Supplementary Table S1). *S. latissima* attached to turf allocated a significantly greater percentage of the thallus biomass to the holdfast as compared to *S. latissima* attached to rock (Fig. 7A, Supplementary Table S2). Kelp attached to turf also had significantly more complex holdfasts, with greater total bifurcations of the haptera within the holdfast as compared to kelp attached to rock (Fig. 7B, Supplementary Table S3). Moreover, kelp attached to turf at 2 depths (2 and 6 m) had significantly slower *in situ* growth rates as compared to kelp attached to rock (Fig. 8, Supplementary Table S4). Percent survival (retention) also was lower for tagged turf-attached kelp (61%) and as compared to tagged rock-attached kelp (81%) over 35 days for the 2 depths combined.

## Discussion

Kelp forests are some of the most biodiverse ecosystems in the world and support primary productivity comparable to that of tropical rain forests^23,24^. However, these marine forests are under threat due to anthropogenic environmental degradation driven by climate change and local stressors such as eutrophication^5,6^. Understanding the primary drivers of kelp decline and the functioning of novel anthropogenic ecosystems that replace kelp forests is of critical importance to management and conservation of marine resources^5^. Here, we documented a substantial decline of kelp abundance at sites at the southernmost extent of kelp forests in the Northwest Atlantic, with order of magnitude decreases in kelp biomass and density in 2017–2018 as compared to baseline data collected in the 1980s. Contemporary biomass values at or near zero are well outside expected natural intra- or interannual variability of *S. latissima* populations^18,19^, suggesting we are observing the loss of kelp forests from this region. Furthermore, we found that once-dominant kelp was replaced by turf-forming macroalgal species, contributing to a global pattern of kelp loss followed by the “rise” of turfs^5^.

The loss of kelp forests at sites in Narragansett Bay is consistent with a multidecadal increase in sea surface temperature above a threshold for survival of *S. latissima* sporophytes and resting-stage gametophytes. This pattern of kelp loss occurred despite a significant decrease in local nutrient loads over the study period. A decrease in ammonium, nitrate, and phosphate concentrations in Narragansett Bay from 1972 to the present is best explained by passage of the Clean Water Act in 1972, which reduced the discharge of pollutants into United States watersheds (https://www.epa.gov/laws-regulations/summary-clean-water-act). This finding indicates a success story for local water management, but with the important implication that local management cannot necessarily protect marine ecosystems against degradation in the face of global climate change. Given current trends in sea temperatures, we predict the continual decline and absence of kelp at the southern extent in the Northwest Atlantic. Thus, despite optimistic forecasts that warm-adapted kelp populations should be the most resilient to climate change impacts^7^, these *S. latissima* populations likely do not have the adaptive capacity to respond to warming sea temperatures at current rates under global climate change. Our findings are consistent, rather, with predictions that distributions of marine species at their upper thermal physiological range limits will contract poleward^25^.

Turf-forming macroalgal species identified as part of our study ranged two phyla (Rhodophyta and Chlorophyta) with diverse evolutionary histories and growth forms (e.g., plumose, filamentous, and finely and coarsely branching), indicating the complex nature of the novel turf-dominated ecosystem. Turf communities are generally comprised of opportunistic, “weedy” species with high tolerance to stress; however no overarching definition of “turf” has been established^11^. Indeed, many studies examining shifts from kelp to turf dominance do not characterize the composition or structure of the turf community outside of general descriptions such as “filamentous” turfs^8^ or “fast-growing” turfs^3^. This exemplifies the extent to which we understand very little about the ecological functioning of these communities, limiting the ability of researchers to generalize their findings across globally disparate turf-dominated ecosystems.

Feedback mechanisms that stabilize the turf-dominated ecosystem may increase the resilience of the ecosystem to a reverse shift back towards kelp dominance, and may indicate the presence of alternative stable states^5^. While we cannot directly test for hysteresis in our dataset, as kelp forests and turf reefs occur under separate environmental conditions of sea temperature, we did identify feedback mechanisms that may stabilize the turf-dominated ecosystem. The presence of such mechanisms suggests that if sea temperature trends were to reverse, the turf-dominated ecosystem could persist beyond the point at which an initial regime shift to turf occurred. We determined that space preemption by turfs results in kelp sporophytes attaching to turf rather than rocky substratum. The turf-attached kelp had lower survival as compared to rock-attached kelp, likely due to significantly weaker attachment strength. Dislodgement of kelp likely occurred during two strong wave events (>3 m significant wave heights) on 17 and 26 April 2018 (Supplementary Fig. S1). This interpretation is consistent with the findings of Burek *et al*. (ref.^9^), who observed lower survival of turf-attached as compared to rock-attached kelp transplanted from wave-protected to exposed sites in Nova Scotia, Canada, and who suggest that this creates a feedback mechanism that stabilizes the turf-dominated ecosystem.

Turf-attached kelp allocate significantly more biomass to the holdfast and have significantly more complex holdfasts than rock-attached kelp^9^. We observed reduced growth rates of turf-attached kelp, which may represent a trade-off in resource allocation from blade growth to holdfast development and extension of haptera to strengthen attachment on a suboptimal substrate. Slower growth of turf-attached as compared to rock-attached kelp may act to limit the reproductive output of kelp in turf-dominated ecosystems, as the size of the sorus will be constrained by blade size^26^. Thus, in combination, increased mortality of turf-attached kelp due to dislodgement, and slower growth of turf-attached individuals should create a feedback loop that stabilizes the turf-dominated ecosystem and limits the reestablishment of kelp.

The mean growth rate of rock-attached kelp in our study (1.2 cm d^−1^) was similar to that observed in the early 1980s by Brady-Campbell *et al*. (ref.^19^). This result suggests that individual-based productivity rates for rock-attached kelp have not changed over an ~40-year period. However, given that approximately half of the kelp were attached to turf and had significantly slower mean growth rates (0.9 cm d^−1^), and kelp biomass also was much lower in 2017 and 2018, population-scale kelp productivity likely has substantially declined. Kelp have a potentially important role in mitigating climate change through the sequestration and storage of carbon that is transported to deep ocean regions as macroalgal detritus^27^. Thus, loss of kelp productivity could act as a positive feedback for climate warming.

A recent study in the Gulf of Maine demonstrated 150 times greater *S. latissima* density at offshore sites (Caches Ledge) than coastal sites, despite thermal stress of kelp in both regions^28^. No information currently exists for offshore kelp forests at the southern limit of *S. latissima* in the Northwest Atlantic. Future research should therefore be focused on determining whether a localized community shift to turf observed on reefs in Narragansett Bay is a region-wide phenomenon, and should investigate management strategies to prevent or reverse the disappearance of kelp forests. In addition to curbing global climate change, local management strategies that involve the removal of turfs to open up space for kelp recruitment should be considered given the ecological feedbacks associated with recruiting to turf documented here. The purple sea urchin *Arbacia punctulata* has the ability to clear small patches in the algal turf and create bare space (authors’ pers. obs.). Interactions between algal turfs, kelp, and grazers on these reefs therefore warrant investigation. Grazing by sea urchins could destabilize a turf-dominated ecosystem if grazed patches are colonized by kelp propagules that survive to reproductive maturity.

## Methods

### Study sites

The study was conducted at 3 sites in Narragansett Bay, Rhode Island: Fort Wetherill (41.478°N, 71.362°W), Land’s End (41.450°N, 71.314°W), and Bass Rock Road (41.405°N, 71.456°W). Narragansett Bay is a 259 km^2^ embayment with a mean depth of 9 m and maximum depth of 60 m and which opens to the south into Rhode Island Sound adjacent Long Island Sound to the west^19^. The sites are moderately wave-exposed based on the angle to which each site is open to exposure from Rhode Island Sound and the criteria discussed in Lewis^29^. Sites span an east (Land’s End) to west (Bass Rock Road) gradient and consist of sloping bedrock and large boulders that grade to sand at ~7 m depth.

### Kelp biomass

We measured kelp biomass at 2 sites (Fort Wetherill and Land’s End) for comparison with available baseline data from the early 1980s^19^. For consistency with the methodology of historical measurements, kelp biomass was measured in June/July at 2 m depth (season and depth of greatest historical kelp density) within n = 6 randomly placed quadrats at each site. While Brady-Campbell *et al*. (ref.^19^) sampled 0.1 m^2^ quadrats, kelp biomass was measured within 0.25 to 1 m^2^ quadrats in our study to minimize zero values at low kelp abundance. For biomass measurements, the wet weight of blotted-dry kelp, destructively sampled from each quadrat, was determined to the nearest 0.01 g with a spring scale to give an estimate of kelp fresh biomass in units of g m^−2^. Given that Brady-Campbell *et al*. (ref.^19^) report their data as dry weight of kelp, for comparison, we converted their measurements to wet weight with the formula for conversion of dry weight (DW) to fresh weight (FW) of *S. latissima*: DW = 0.113 FW (r^2^ = 0.970)^30^. No individuals of *L. digitata* were observed in the quadrats at any site.

### Kelp density

We compared kelp density at Fort Wetherill in 2017–18 to baseline data collected in 1980–81 by Brady-Campbell *et al*. (ref.^19^). To measure the density of kelp, divers counted kelp in belt transects at 2 to 6 m depth (n = 2–3 transects) on 1 Dec 2017, 20 Dec 2017, 9 Feb 2018, and 23 Mar 2018, and in quadrats placed randomly at 2 to 6 m depth (n = 20 1-m^2^ quadrats) on 20 Jun and 30 Nov 2018. Belt transects were used on the earlier dates due to very low (near zero) kelp density, which required a large sampling area to minimize zero values. On each sampling date, divers counted and measured the blade lengths of all kelp encountered within the belt transects or quadrats. Blade lengths were used to produce size-frequency distributions. The width of the transect was 2 m, but the length varied throughout the season (ranging from 3 to 30 m) to accommodate changing kelp density, with larger transects used on lower density dates. The total number of individuals observed on each sampling date ranged from 48 to 139. Transects and quadrats were pooled to determine overall density. In all density surveys conducted at Fort Wetherill, only a single individual of *L. digitata* was observed (20 Dec 2017).

We observed both a winter and spring recruitment pulse of *S. latissima* at Fort Wetherill (Supplementary Fig. S2). In early December 2017, all kelp observed along belt transects were <70 cm in blade length, indicating recently recruited individuals. By March 2018 individuals with blade lengths up to 180 cm were observed, indicating somatic growth of winter recruits. A second pulse of recruits with blade lengths <70 cm was observed during sampling in June 2018 (Supplementary Fig. S2). Individuals with blade lengths >70 cm were rare on 20 Jun (7 out of a total of 139 individuals observed), indicating low survival of the winter recruits.

### Percent cover of turf

To assess the abundance of turf algae at sites in Narragansett Bay, we measured the percent cover of turf-forming macroalgae from video data collected at 2 to 6 m depth at 3 sites (Fort Wetherill, Land’s End, and Bass Rock Road). In late June or early July 2018, divers swam along 30 m transects parallel to shore at each depth and site with a GoPro Hero 4 camera positioned ~1 m above bottom. For each video, photoquadrats were extracted every 3 m (n = 10 per video) and pooled over depths to calculate percent cover of turf (n = 20 images per site/date). For the image analysis, a 50 × 50 cm grid was superimposed on the photoquadrat using the width of the transect tape to set the scale. Percent cover of turf was then estimated by assessing the presence or absence of turf on each section of the grid^3^. Occasionally, poor resolution in the photo rendered a section of the image unreadable. In such cases the section was discarded, and percent cover was estimated based on the remaining part of the photoquadrat. Image analysis was conducted with the program Image J.

### Turf species assemblage

To characterize the turf-forming macroalgal community, we determined the identity of macroalgal species at Fort Wetherill from destructive sampling of n = 3–6 0.1 m^2^ quadrats placed haphazardly ~5 m apart at 4 m depth on 1 Dec 2017, 9 Feb 2018, and 1 May 2018. For the turf collection, a stainless-steel scraper was used to release all macroalgae from the substratum and divers manually collected the macroalgae and placed it in a mesh bag (5 mm diameter mesh). Species were identified under a dissecting microscope in the laboratory using guides for algae identification for the East Coast of the United States^31,32^ and Algaebase.org. Wet weight of each turf species was measured to the nearest 0.01 g to assess the biomass contribution of each species.

### Environmental drivers

We acquired weekly sea surface temperature data from the Narragansett Bay Plankton Time Series at the Graduate School of Oceanography, University of Rhode Island (ref.^33^ and https://web.uri.edu/plankton/). Data were analyzed over a 58-year period from 1959 to 2017 to determine the total number of weeks in each year with sea temperatures greater than or equal to 7 thresholds between 14 and 26 °C (2 °C increments). The thermal integral of weeks with sea temperatures ≥22 °C was then calculated for each year as the sum of weekly temperature for weeks with temperatures ≥22 °C (degree-week). Nutrient data also were acquired from the Narragansett Bay Plankton Time Series and analyzed from 1972 to 2018 to test for temporal changes in ammonium, nitrate, and phosphate concentrations indicative of local nutrient loading.

### Kelp recruitment onto turf

The proportion of kelp recruiting onto turf-forming macroalgae versus rocky substratum was determined in belt transects surveyed for kelp density at Fort Wetherill (see Kelp density). To determine the proportion of kelp attached to turf-forming macroalgae, divers noted the number of kelp individuals in transects with holdfasts attached to turf or rock. The percentage of kelp recruited to turf was calculated as the number of individuals on turf divided by the total number of individuals observed on either substrate.

### Force to detach kelp from turf and rock

Observations that kelp were commonly recruited onto turf-forming macroalgae prompted a study to examine the attachment strength of kelp attached to rocky substrate versus turf to determine the risk of detachment by wave forces for these 2 groups of kelp. We predicted that kelp attached to turf would require less force to detach than kelp attached to rocky substrate, as kelp appeared loosely attached to the turf. On 20 Dec 2017 (n = 15 rock, and 12 turf), 9 Feb 2018 (n = 5 rock, and 6 turf), and 22 May 2018 (n = 8 rock, and 9 turf), divers used a resettable modified dynamometer^34,35^ consisting of a spring scale attached to an elastic nylon cord (to avoid tearing the holdfast) to test attachment strength of kelp attached to rock and turf. Kelp were selected haphazardly within an ~200 m^2^ area of reef at 4 m depth at Fort Wetherill. The scale was attached to kelp by threading the nylon through the holdfast (attached to rock or turf) and secured with a loose knot. Kelp were then pulled perpendicular to the substrate until the kelp (including holdfast) was dislodged or holdfast failure occurred (cord pulls through the material of the holdfast). The indicator deflection distance on the spring scale was measured with calipers and the attachment strength was determined by multiplying the indicator deflection distance by the known spring constant^34,35^.

Blade lengths of kelp detached from the substrate on 20 Dec 2017 were significantly different among turf- and rock-attached individuals, with turf-attached individuals having a significantly smaller mean blade length (Supplementary Fig. S3). To ensure that force to detach measurements were not biased by kelp blade length (i.e. kelp age/size), we plotted the force to detach as a function of the length of individuals within each group (turf- and rock-attached). We found no relationship between force to detach and blade length (Supplementary Fig. S3).

### Kelp holdfast morphology on turf and rock

To quantify potential differences in holdfast morphology, holdfasts of kelp attached to rocky substrate and turf-forming macroalgae were haphazardly collected at Fort Wetherill from within an ~200 m^2^ area of reef at 4 m depth on 9 Feb (n = 10 rock, and 9 turf), 12 Apr (n = 10 rock, and 10 turf), and 1 May 2018 (n = 10 rock, and 9 turf). The blade and stipe were removed from the holdfast of the kelp and each section was weighed to the nearest 0.01 g to determine the percentage of the total biomass (%) occupied by the holdfast. Additionally, the total number of bifurcations of haptera within each holdfast was measured to determine holdfast complexity for kelp collected at 4 m depth at Fort Wetherill on 23 Mar (n = 10 rock, and 9 turf), 12 Apr (n = 10 rock, and 10 turf), and 1 May 2018 (n = 10 rock, and 9 turf).

### Kelp growth rates on turf and rock

To examine a potential trade-off associated with the allocation of greater energy to the holdfast of turf-attached versus rock-attached kelp, we examined growth rates of kelp attached to each substrate type in the field. Growth rate was assessed with the hole-punch method, whereby a hole is punched in the blade at a known distance above the meristematic tissue and migration of the hole is measured through time as an indication of blade growth^17^. Rock-attached and turf-attached individuals that were ~100 cm in blade length were selected haphazardly within ~200 m^2^ of reef at each of 2 m (n = 8 rock-attached and 10 turf-attached) and 6 m (n = 4 rock-attached and 11 turf-attached) depth. On 12 Apr 2018, individuals were hole-punched 10 cm above the meristematic tissue and then tagged with a plastic numbered tag for individual identification. Divers determined survival (retention) of individuals after 35 days (12 Apr to 17 May), and for surviving individuals, measured the migration distance of the hole on each individual to the nearest cm with a plastic measuring tape. Apr to May is the period over which Brady-Campbell *et al*. (ref.^19^) observed maximal growth of kelp at Fort Wetherill in 1980 and 1981. Egan and Yarish (ref.^16^) also observed maximal growth of *S. latissima* in Long Island Sound in Apr and May.

### Statistical analysis

Two-way ANOVAs were used to examine the effect of substrate (fixed factor, two levels: rock and turf) and month (random factor, three levels: ranging from Dec to May) on 1) the force (N) required to detach *S. latissima*, 2) the percentage of biomass allocated to the holdfast of *S. latissima*, and 3) the total number of bifurcations within the holdfast of *S. latissima*. Two-way ANOVA also was used to examine the effect of substrate (fixed factor, two levels: rock and turf) and depth (random factor, two levels: 2 and 6 m) on the growth rate (cm d^−1^) of *S. latissima* over 35 d from 12 Apr to 17 May 2018. Assumptions of normality were examined with the Shapiro-Wilk test (α = 0.05). Assumptions of homogeneity of variance were examined with the Brown–Forsythe test (α = 0.05). Data were analyzed with Statistica 13.2 (StatSoft) and SigmaStat 4.0 (Systat).

## Supplementary information

Supplementary Information

## Data Availability

All data analyzed are included in this article (and its Supplementary Information Files).

## References

[1] Moy FE, Christie H (2012). Large-scale shift from sugar kelp (*Saccharina latissima*) to ephemeral algae along the south and west coast of Norway. Marine Biology Research.

[2] Steneck RS, Leland A, McNaught DC, Vavrinec J (2013). Ecosystem flips, locks, and feedbacks: The lasting effects of fisheries on Maine’s kelp forest ecosystem. Bulletin of Marine Science.

[3] Filbee-Dexter K, Feehan CJ, Scheibling RE (2016). Large-scale degradation of a kelp ecosystem in an ocean warming hotspot. Marine Ecology Progress Series.

[4] Wernberg T (2016). Climate-driven regime shift of a temperate marine ecosystem. Science.

[5] Filbee-Dexter K, Wernberg T (2018). Rise of turfs: A new battlefront for globally declining kelp forests. BioScience.

[6] Krumhansl KA (2016). Global patterns of kelp forest change over the past half-century. Proceedings of the National Academy of Sciences.

[7] Muth AF, Graham MH, Lane CE, Harley CDG (2019). Recruitment tolerance to increased temperature present across multiple kelp clades. Ecology.

[8] Gorman D, Connell SD (2009). Recovering subtidal forests in human-dominated landscapes. Journal of Applied Ecology.

[9] Burek KE, O’Brien JM, Scheibling RE (2018). Wasted effort: recruitment and persistence of kelp on algal turf. Marine Ecology Progress Series.

[10] O’Brien JM, Scheibling RE (2018). Turf wars: competition between foundation and turf-forming species on temperate and tropical reefs and its role in regime shifts. Marine Ecology Progress Series.

[11] Connell SD, Foster MS, Airoldi L (2014). What are algal turfs? Towards a better description of turfs. Marine Ecology Progress Series.

[12] Scheffer M, Carpenter S, Foley JA, Folke C, Walker B (2001). Catastrophic shifts in ecosystems. Nature.

[13] Gerard VA, Du Bois KR (1988). Temperature ecotypes near the southern boundary of the kelp *Laminaria saccharina*. Marine Biology.

[14] Lee JA, Brinkhuis BH (1986). Reproductive phenology of *Laminaria saccharina* (l.) Lamour (Phaeophyta) at the southern limit of its distribution in the Northwestern Atlantic Ocean. Journal of Phycology.

[15] Lee JA, Brinkhuis BH (1988). Seasonal light and temperature interaction effects on development of *Laminaria saccharina* (Phaeophyta) gametophytes and juvenile sporophytes. Journal of Phycology.

[16] Egan B, Yarish C (1990). Productivity and life history of *Laminaria longicruris* at its southern limit in the Western Atlantic Ocean. Marine Ecology Progress Series.

[17] Parke M (1948). Studies on British Laminariaceae. I. Growth in *Laminaria saccharina* (L.) Lamour. Journal of the Marine Biological Association of the United Kingdom.

[18] Chapman ARO (1984). Reproduction, recruitment and mortality in two species of *Laminaria* in southwest Nova Scotia. Journal of Experimental Marine Biology and Ecology.

[19] Brady-Campbell MM, Campbell DB, Harlin MM (1984). Productivity of kelp (*Laminaria* spp.) near the southern limit in the northwestern Atlantic Ocean. Marine Ecology Progress Series.

[20] Rice E, Dam HG, Stewart G (2015). Impact of climate change on estuarine zooplankton: surface water warming in Long Island Sound is associated with changes in copepod size and community structure. Estuaries and Coasts.

[21] Redmond, S. Effects of increasing temperature and ocean acidification on the microstages of two populations of *Saccharina latissima* in the Northwest Atlantic. MSc Thesis (2013).

[22] Harlin MM, Rines HM (1993). Spatial cover of eight common macrophytes and three associated invertebrates in Narragansett Bay and contiguous waters, Rhode Island, USA. Botanica Marina.

[23] Mann KH (1973). Seaweeds: their productivity and strategy for growth. Science.

[24] Dayton PK (1985). Ecology of kelp communities. Annual Review of Ecology and Systematics.

[25] Harley CD (2012). Effects of climate change on global seaweed communities. Journal of Phycology.

[26] Demes KW, Graham MH (2011). Abiotic regulation of investment in sexual versus vegetative reproduction in the clonal kelp *Laminaria sinclairii* (Laminariales, Phaeophyceae). Journal of Phycology.

[27] Krause-Jensen D (2018). Sequestration of macroalgal carbon: the elephant in the Blue Carbon room. Biology Letters.

[28] Witman JD, Lamb RW (2018). Persistent differences between coastal and offshore kelp forest communities in a warming Gulf of Maine. Plos ONE.

[29] Lewis, J. R. *The ecology of rocky shores*. 323 pp (English Universities Press, London, 1964).

[30] Gevaert F (2001). Carbon and nitrogen content of *Laminaria saccharina* in the eastern English Channel: biometrics and seasonal variations. Journal of the Marine Biological Association of the United Kingdom.

[31] Hillson, C. J. *Seaweeds: a color-coded, illustrated guide to common marine plants of the East Coast of the United States*. (Penn State Press, 1990).

[32] Villalard-Bohnsack, M. Illustrated key to the seaweeds of New England. *Rhode Island Natural History Survey* (2003).

[33] Smayda, T. J. & the Bunker C community. Narragansett Bay Plankton Time Series. Graduate School of Oceanography, *URI. Data available at: NABATS.org* (1959–1997).

[34] Carrington E (1990). Drag and dislodgment of an intertidal macroalga: consequences of morphological variation in *Mastocarpus papillatus* Kützing. Journal of Experimental Marine Biology and Ecology.

[35] Bell EC, Denny MW (1994). Quantifying “wave exposure”: a simple device for recording maximum velocity and results of its use at several field sites. Journal of Experimental Marine Biology and Ecology.

