# Supplementary material for: Ecological feedbacks stabilize a turf-dominated ecosystem at the southern extent of kelp forests in the Northwest Atlantic
Source: Sci Rep. 2019 May 8;9:7078. doi: 10.1038/s41598-019-43536-5 (PMC6506546; doi:10.1038/s41598-019-43536-5)
Supplement: Supplementary file 1 — Supplementary Information [file 41598_2019_43536_MOESM1_ESM.pdf]

Supplementary Information for

Ecological feedbacks stabilize a turf-dominated ecosystem at the southern extent of kelp forests in the Northwest Atlantic

Colette J. Feehan, Sean P. Grace, and Carla A. Narvaez

PDF contains:

Supplementary Table S1–S4

Supplementary Fig. S1–S3

Supplementary Table S1. Two-way ANOVA of the effect of substrate (fixed factor, two levels: rock and turf) and month (random factor, three levels: Dec, Feb, and May) on the force (N) required to detach kelp *Saccharina latissima* at 4 m depth at Fort Wetherill. **Bold** values are significant at α = 0.05.

**Source of Variation**	**df**	**MS**	***F***	**p**
Substrate	1	19560.692	134.582	**<0.001**
Month	2	9673.02	111.57	**<0.001**
Substrate × Month	2	86.702	0.597	0.555
Error	48	145.344		

Supplementary Table S2. Two-way ANOVA of the effect of substrate (fixed factor, two levels: rock and turf) and month (random factor, three levels: Feb, Apr and May) on the percentage of biomass allocated to the holdfast (%) of kelp *Saccharina latissima* at 4 m depth at Fort Wetherill. **Bold** values are significant at α = 0.05.

**Source of Variation**	**df**	**MS**	***F***	**p**
Substrate	1	0.0425	25.985	**<0.001**
Month	2	0.0359	10.497	**0.008**
Month × Substrate	2	0.00342	2.092	0.137
Error	40	0.00163		

Supplementary Table S3. Two-way ANOVA of the effect of substrate (fixed factor, two levels: rock and turf) and month (random factor, three levels: Mar, Apr and May) on the total number of bifurcations within the holdfast of kelp *Saccharina latissima* at 4 m depth at Fort Wetherill. **Bold** values are significant at α = 0.05.

**Source of Variation**	**df**	**MS**	***F***	**p**
Substrate	1	321.038	101.429	**<0.001**
Month	2	8.894	5.47	**0.024**
Substrate × Month	2	1.626	0.514	0.601
Error	52	3.165		

Supplementary Table S4. Two-way ANOVA of the effect of substrate (fixed factor, two levels: rock and turf) and depth (random factor, two levels: 2 and 6 m) on the growth rate (cm d^{-1}) of kelp *Saccharina latissima* at Fort Wetherill over 35 d from 12 Apr to 17 May 2018. **Bold** values are significant at α = 0.05.

**Source of Variation**	**df**	**MS**	**F**	**p**
Substrate	1	0.352	6.826	**0.0182**
Depth	1	0.231	4.620	**0.0264**
Substrate × Depth	1	0.05	0.974	0.338
Error	17	0.052		

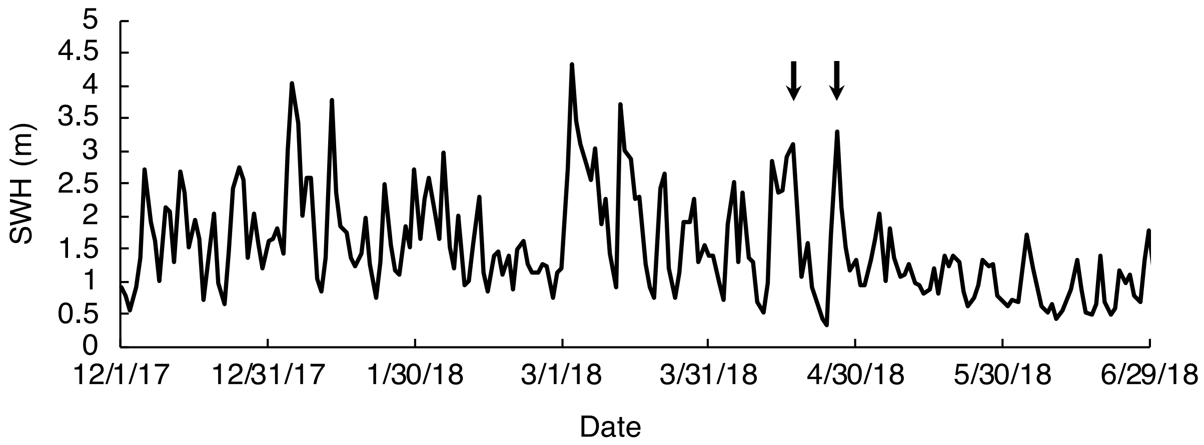

Supplementary Fig. S1. Significant wave height (SWH, m) from NOAAs National Buoy Data Center for Station 44097, Block Island, RI. Arrows indicate relatively large SWH (>3 m) observed during the kelp tagging experiment.

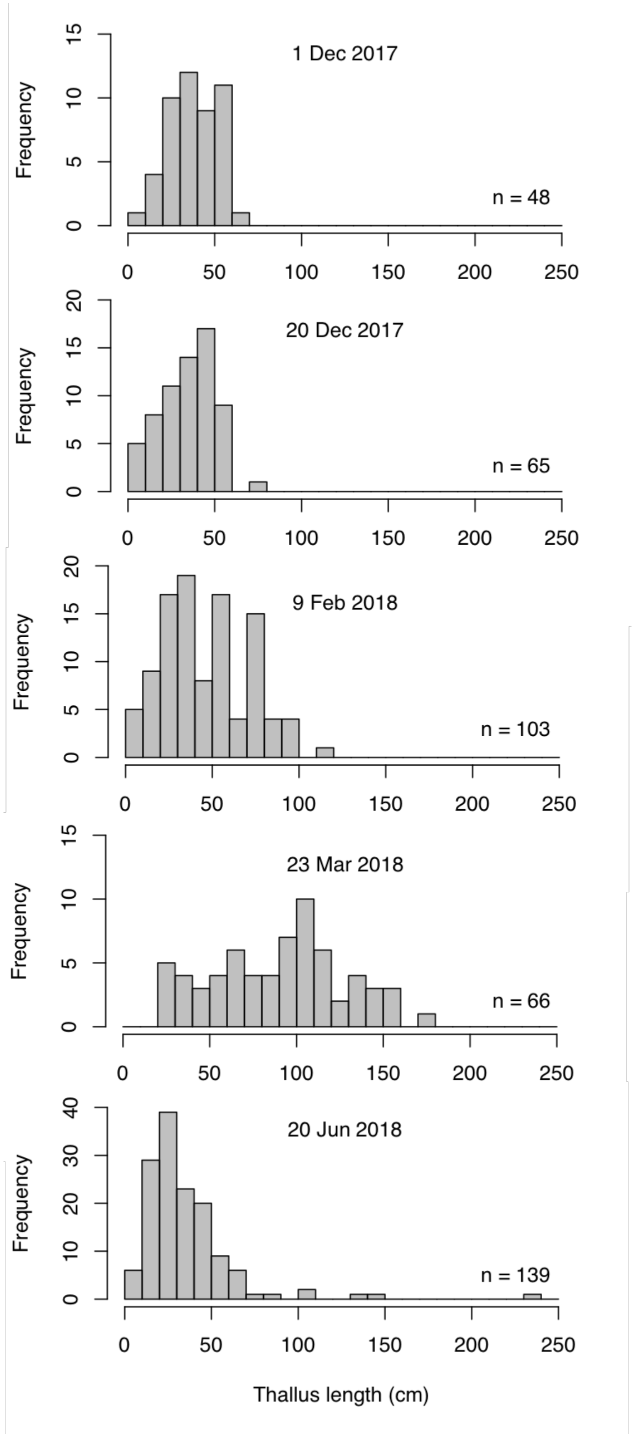

Supplementary Fig. S2. Size frequency distributions of kelp *Saccharina latissima* blade lengths (cm) at Fort Wetherill in winter 2017 to spring 2018.

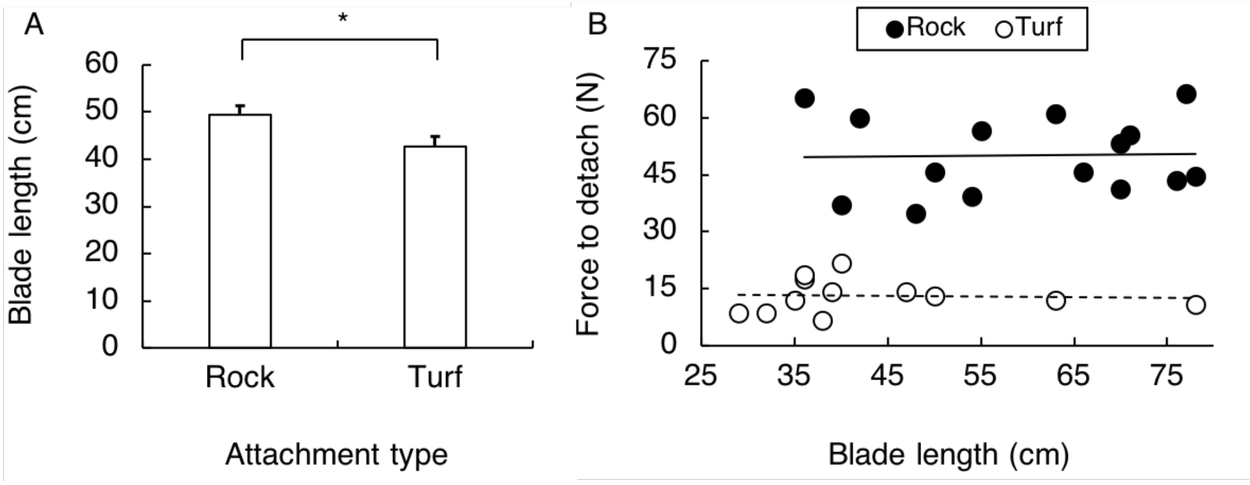

Supplementary Fig. S3. A. Mean (+SE) blade length of kelp *Saccharina latissima* attached to rocky substratum and turf-forming macroalgae in December 2017 at 4 m depth at Fort Wetherill (asterisk indicates significant difference at $\alpha = 0.05$ based on a 2-sample t-test). **B.** Force (N) to detach rock- and turf-attached kelp as a function of blade length (cm) (n = 15 rock, and 12 turf). Lines indicate non-significant relationships at $\alpha = 0.05$: Force = 0.0229×Length + 48.697, R^2 = 0.001 (Rock); Force = -0.0195×Length + 14.02, R^2 = 0.004 (Turf).
